# Top-Down Proteomics and Farm Animal and Aquatic Sciences

**DOI:** 10.3390/proteomes4040038

**Published:** 2016-12-21

**Authors:** Alexandre M.O. Campos, André M. de Almeida

**Affiliations:** 1 CIIMAR—Interdisciplinary Centre of Marine and Environmental Research, University of Porto, 4450-208 Matosinhos, Portugal; acampos@ciimar.up.pt; 2 Clinical Department, RUSVM—Ross University School of Veterinary Medicine, PO Box 334, Basseterre, Saint Kitts and Nevis

**Keywords:** farm animal proteomics, aquatic sciences proteomics, top-down proteomics

## Abstract

Proteomics is a field of growing importance in animal and aquatic sciences. Similar to other proteomic approaches, top-down proteomics is slowly making its way within the vast array of proteomic approaches that researchers have access to. This opinion and mini-review article is dedicated to top-down proteomics and how its use can be of importance to animal and aquatic sciences. Herein, we include an overview of the principles of top-down proteomics and how it differs regarding other more commonly used proteomic methods, especially bottom-up proteomics. In addition, we provide relevant sections on how the approach was or can be used as a research tool and conclude with our opinions of future use in animal and aquatic sciences.

## 1. Introduction

Proteomics may be defined as the study of the proteome in a given cell, tissue, organ, organism, or even at the population or multi-population level. Initiated approximately two decades ago with the advent of protein gel electrophoresis and mass spectrometry instruments, proteomics is today a well-established field of research with numerous applications in very diverse sciences such as plant sciences, human medicine, bacteriology, and other fields. The use and adoption of proteomics in animal, veterinary, and aquatic sciences have been relatively slow when compared to other areas of research. Nevertheless, the last 10 years have witnessed a substantial increase in proteomics research in these areas [1,2]. In fact, numerous examples are available in the literature and include, for instance, research on meat science [3,4], colostrum and milk science [5], the creation of vaccines for tickborne diseases [6], the establishment of welfare and stress biomarkers [7], diverse uses in aquaculture [8], or the establishment of pollution markers in bivalves [9].

The majority of the species studied in farm animal and aquatic sciences research are termed non-model species. Working with such species poses several inconveniences, particularly related to their relatively low representation in protein databases [10]. In fact, with the exception of cattle [11], pig [12,13], and rabbit [14], other species’ representation in search databases is extremely low. The characteristics of these databases bring technical limitations, particularly at the level of protein identification that must be established through homology searches [2]. It also brings some limitations to the choice of proteomic methods that can be used in Animal and Aquatic Science Proteomics (AASP). In fact, in these fields of research, two-dimensional gel electrophoresis (2DE) has been termed “the workhorse” for proteomics research [2]. Numerous examples may be found in the literature on AASP research using 2DE, including single stain gels [15,16,17,18] and 2DE-DIGE (difference gel electrophoresis) [19]. Additional alternative gel techniques such as blue native polyacrylamide (PAGE) gels, particularly suitable for membrane proteomics, and the study of protein complexes and protein–protein interactions [20], such as those in the mitochondria [21], have also been used. More recently, with the improvement of databases and the relative decrease on instrumentation prices, gel-free techniques such as iTRAQ (isobaric tag for relative and absolute quantitation) or label-free proteomics have increasingly become more popular and widespread in the AASP research community. Examples include the use of iTRAQ to study the wool proteome [22] and colostrum protein uptake [23], or to use label-free proteomics to study the goat mammary gland [24] or marine mussel (*Mytilus edulis*) response to salinity [25]. The previously mentioned techniques are termed bottom-up proteomics (BUP) (see next section). However, in recent years, top-down proteomics (TDP) have also been established and, as in other fields of research, their use in AASP is still very incipient. Nevertheless, it is a technique that is available and that in the coming years will likely become more and more common in AASP research.

**Figure 1:**
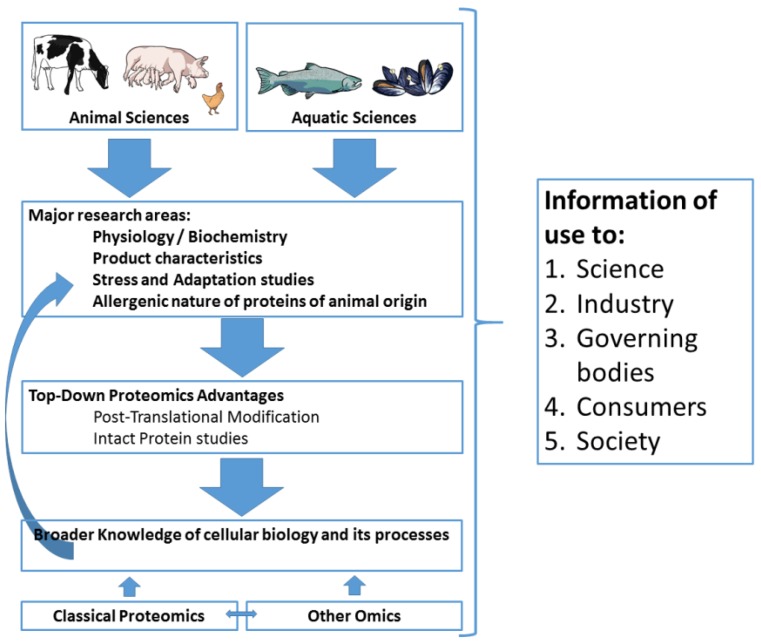
Interaction of top-down proteomics with animal and aquatic sciences illustrating the advantages of including the technique in research programs that include classical proteomics and other omics (Transcriptomics, Lipidomics, or Metabolomics).

This article is dedicated to TDP in the framework of AASP research and includes both a review and an opinion perspective, intended for individuals in animal/veterinary and aquatic sciences, particularly those already using proteomics and/or contemplating the use of TDP. The rationale behind this article is illustrated in Figure 1 and shows major areas of research in farm animal and aquatic sciences and how they might interact with top-down proteomics. After this brief introduction, Section 2 will focus on the characteristics of a TDP approach, its use, required instrumentation, and advantages and drawbacks inherent to this approach. Section 3 and Section 4 will focus on specific examples where TDP has been successfully used in the fields of animal/veterinary sciences and aquatic sciences, respectively, in order to illustrate the advantages inherent to this approach. Finally, in Section 5, we will conclude with our personal views on the usefulness of TDP to these two exciting fields.

## 2. Basis of Top-Down Proteomics

Mass spectrometry (MS)-based proteomics is typically carried out by first digesting a protein mixture into short peptides (commonly up to 3 kDa mass) with a protease, then analyzing the peptide mixture by MS [26]. This strategy is known as “bottom-up” (BUP) or “shotgun” proteomics and is the current strategy for carrying out large-scale analysis of proteomes. In contrast, TDP typically regards the analysis of proteoforms by direct analysis of intact proteins by MS, without previous proteolytic digestion [27]. TDP is also attained with 2DE to resolve proteoforms in combination with MS. Another MS strategy has been developed, called middle-down proteomics (MDP), which refers to the analysis of incomplete digested proteins or protein fragments [26]. This strategy aims to improve bottom-up approaches and to bring more protein structure information to MS data.

The development of TDP is associated with the implementation of MS-based approaches. These purified intact proteins are ionized by electrospray ionization (ESI) and trapped in a Fourier-transform ion cyclotron resonance (Penning trap) (FTICR) [28] or quadrupole ion trap (Paul trap) mass analyzers [29]. Fragmentation of intact proteins for tandem mass spectrometry is then accomplished by electron-capture dissociation (ECD) [30] or electron-transfer dissociation (ETD) [31]. The electron-based methods for ion fragmentation are highly convenient, since cleaved proteins and large peptides in many more backbone positions than gas-based approaches, resulting in an improved capacity for sequence coverage and, therefore, localization of post-translational modifications (PTMs) such as phosphorylation, glycosylation, acetylation, proteolysis, and oxidized species [27,32]. Also, ECD can retain PTMs that are more labile during the original ionization process, herein contrasting with collisional induced dissociation (CID) of tryptic peptides that often leads to the loss of certain chemical groups of amino acids [33,27]. An example of such labile PTM is gamma-carboxylation of glutamic acid residues, a post-translational modification of several proenzymes of the blood coagulation cascade [33]. Recently, however, TDP has changed from the abovementioned MS-centric definition towards a broader definition with the resolution of intact proteoforms as the most characteristic trait, including, for instance, the interfacing of 2DE with mass spectrometry.

The analytical power and sensitivity of TDP makes the approach highly suitable for characterization of protein PTMs and identification of families and expression ratios of highly related genes encoding protein sequences with high identity, protein polymorphisms, and alternative splicing isoforms [27]. In this regard, BUP approaches are not efficient in retrieving such information from the analysis of peptides produced by proteolysis of complex protein samples, making the distinction of peptides that are common to several isoforms from isoform-specific peptides almost impossible [27].

High-sensitive Fourier-transform MS (FTMS) instruments and an orbitrap are required to carry out TDP. The complexity of MS data generated from TDP approaches requires specific tools to be developed for interpretation and database searching. ProSight is an algorithm specifically developed for this purpose. ProSight specifically uses a candidate expansion method referred to as “shotgun annotation”, combining data from diverse sources regarding potential mass differences—such as polymorphisms, alternate splicing, and PTMs—to assist protein characterization. The user can optionally control how much biological variability should be searched [34,35]. Other software dedicated to TDP data acquisition and interpretation include MASH Suite Pro (http://crb.wisc.edu/yinglab/software.html) [36] and Autopilot (http://nrtdp.northwestern.edu/technology-research-development/trd-3/) [37].

Sample fractionation prior to MS analysis is an important step in TDP. Sample fractionation has been carried out by preparative gel electrophoresis, or by 2D chromatography combining anion exchange [38], capillary isoelectric focusing [39] or chromatofocusing [40] with reversed-phase liquid chromatography (RPLC). However, the lack of resolving power and separation efficiency of these methods has been confining TDP mostly to the analysis of individual intact proteins or simple protein mixtures [41]. Two-dimensional gel electrophoresis is one of the most effective methods to fractionate complex protein mixtures and proteoforms, and for this reason it could also be considered a top-down analytical approach. Thus, 2DE, in combination with MS-based protein identification methods, allows for the resolution of thousands of intact protein species to achieve the molecular identities of proteins, including isoforms and PTMs [42].

TDP may be independent of protein digestion, thereby some of the limitations associated with digestion-dependent procedures and to BUP approaches (i.e., insufficient tryptic peptides generated for analysis, incomplete recovery of peptides following proteomic digestion that results in limited sequence coverage) can be surpassed, enabling unique information to be retrieved about the state of a protein (e.g., the presence of sequence variations arising from point mutations, alternative splicing events, or PTMs) [43,44].

Another advantage may arise with the analysis of intact proteins, in which the abundance of the protein forms determined directly from intact proteins will be less susceptible to instrumental biases than are their small peptide counterparts from peptide-based approaches [45].

Protein identification and proteoform characterization in the TDP approaches suffer from a dynamic range challenge, where the same highly abundant species are repeatedly fragmented [41]. In this regard, TDP does not have the same performance as BUP to undertake large-scale proteomic analyses, which require an effective intact protein fractionation method integrated with tandem mass spectrometry [35]. It is anticipated that future advances in TDP will target primarily new protein separation methods, but also the development of faster mass spectrometers and informatics tools to process complex MS data sets [46].

TDP proteomics has several important advantages that make it extremely useful for the AASP research community. The study of intact proteins and PTM characterization are very important to understand specific biological processes of interest to animal and aquatic sciences. In the next two sections, we will illustrate how TDP has been used in animal and aquatic sciences, highlighting the usefulness of the technique and the possibilities it may open in the future.

## 3. Top-Down Proteomics in Animal Sciences

In the last two decades, the use of proteomics in animal sciences has been rapidly expanding. Its applications are varied and include aspects as different as classical farm animal physiology to product (meat, dairy, etc.) characterization and detection of frauds in foods of animal origin [2]. The majority of the proteomics research in farm animal sciences has been done mostly through the use of gel-based techniques and, more recently, BUP-based shotgun/gel-free proteomics, whereas the use of TDP has been extremely limited. Several factors contribute to this reality, particularly the lack of awareness of the possibilities the technique can bring to the animal science research community, high instrument costs, and limited availability of instrumentation. However, the use of TDP seems to be expanding, and over the last half-decade several examples of its use may be found in the literature concerning farm animals. Therefore, it is likely that, similar to other proteomic techniques like 2DE/DIGE, iTRAQ, or label-free proteomics, its use will expand in the coming years.

Zhang and co-workers [32], conducted one of the earliest studies using TDP in swine. These authors used pigs as a model for cardiovascular research in humans, characterizing the modifications in swine troponin by using high-resolution TDP in conjugation with immunoaffinity chromatography purification. The study demonstrated that swine cardiac troponin I (cTnI) affinity-purified from domestic pig hearts was N-terminally acetylated and phosphorylated. While the study clearly demonstrated the unique power of TDP in the characterization of protein modifications in a farm animal species, the results contributed valuable information not just to the *Sus scrofa* species but also by analogy to humans. The same group has more recently used TDP to study tropomyosin (TPM) isoforms and PTMs that regulate TPM function in swine skeletal muscle [47]. The authors characterized the TPM sequence and localized PTMs such as acetylation, phosphorylation, and amino acid polymorphisms. Furthermore, the authors were able to characterize a TPM isoform that could be added to the existing swine TPM sequences that proved to be identical to an already characterized mouse isoform. In 2016, the same research group used a similar approach to characterize TPM isoforms in the skeletal muscle of humans and rats by comparison to swine [48]. The authors characterized major isoforms for the three species and used MS methodology to identify the sequences. The results revealed muscle-type-specific differences in the abundance of unmodified and modified TPM isoforms in rat and human skeletal muscles. The works by these authors clearly demonstrated the importance of TPM for the thorough characterization of isoforms of muscle proteins and PTMs and their role on muscle physiology and muscle-related disease. The future is bright for applications in the field of peptidomics and meat science (e.g., meat contamination/adulteration) [4]. TDP has also been used to evaluate homogenized tissue followed by depletion of large proteins and subsequent application of capillary LC–MS in order to characterize lower molecular weight (MW) (500–5000 Da) species in tissue [49]. The authors of this review have surveyed several sheep (*Ovis aries*) tissues (heart, lung, liver, kidney, and spleen) and demonstrated the reproducibility of the method and its usefulness to characterize low molecular mass proteins or large peptides.

In the last two years, TDP has also been used to characterize epididymal sperm maturation [50]. Boar spermatozoa isolated from four different epididymal regions (immature to mature stage) were analyzed combining differential and quantitative MALDI-TOF profiling for whole cells or subcellular fractions. Whole cells and subcellular fractions were combined with TDP directed at specific proteins in order to identify endogenous biomolecules and establish the importance of protease activity in sperm maturation and fertility. The same group studied chicken semen (spermatozoa and seminal plasma) peptidome/proteome, linking it to a molecular phenotype related to sperm quality. The authors used three quantitative strategies (fluid and intact cells MALDI-MS, SDS-PAGE combined to LC-MS/MS with spectral counting, and XIC (extracted ion chromatograms) methods). The combination of approaches allowed the characterization of specific profiles per male and the identification of 16 biomolecules of relevance to avian fertility. The authors also identified several proteins involved in different biochemical pathways (oxidoreduction mechanisms, energy processes, proteolysis, and protein localization) with a relevant role in rooster fertility. The same group recently conducted a complimentary study [51] in which a larger standardized experimental procedure was designed for the development of a fertility-predictive mathematical model based on sperm cell MALDI-TOF MS profiles acquired through a fast, automated method. The authors determined that an intact cell MALDI-TOF MS-based method showed high diagnostic accuracy in identifying fertile/subfertile males in a large male population of known fertility from two distinct genetic lineages (meat production and egg laying). Additionally, identification of 40% of the *m*/*z* peaks observed in the sperm MS profiles through a top-down high-resolution protein identification analysis contributed to the study of proteomic markers in avian fertility in a model that can possibly be translated to other species. These studies demonstrated the importance of combining TDP with other approaches to successfully study male fertility, a process of capital importance in animal husbandry and production.

The mentioned studies have in common the use of TDP to study specific protein characteristics or protein roles in specific physiological processes, such as muscle contractility or sperm maturation. They also show the potential for the combination of top-down proteomics with more classical and standardized proteomic methods—revealing the high complimentarily nature of this approach and the plethora of biological processes of specific interest to the animal science research community—that this technique may bring.

## 4. Top-Down Proteomics in Aquatic Sciences

The use of proteomics is having a remarkable growth in aquatic sciences due to the increasing need to attain more information from the biological systems in aquatic environments. Interest at the level of gene and protein function will provide a more comprehensive understanding of a species’ biology and its response/adaptation to the environment. The main areas of research that have adhered to this technology have been aquaculture for improving fish health and nutrition, welfare assessment and stress reduction, diseases and the use of antibiotics and vaccines, aquatic toxicology, and the identification of major environmental threats and the mode of action (MOA) of water contaminants [52,53,54]; developmental biology and reproduction [55,56,57]; and physiology and immune mechanisms in selected wildlife and commercial species [58,59,60,61]. The conventional proteomic techniques such as quantitative 2DE and 2D-DIGE in combination with MS have been the most used to address specific proteomic questions raised in aquatic sciences. There is, however, a recent adhesion to high-throughput strategies based on MS for the analysis of the proteome. BUP approaches are now well established in aquatic sciences, as evidenced by the growing number of recent projects of full RNA and DNA sequencing, thus, enabling the access to new genomic information from a considerable number of aquatic species, including fish, crustaceans, bivalve mollusks and lower-order animals, aquatic model organisms, and farmed species. BUP has been particularly relevant to the discovery of novel biomarkers for environmental monitoring and quality control of fish food [62,63]; identification of new biomarkers in sentinel organisms (clams) exposed to inorganic environmental stressors such as copper, arsenic, cadmium and pharmaceuticals [25,64,65]; investigation of the molecular pathogenesis of virus-associated shrimp and fish diseases [66] and susceptibility of two oyster species to a protozoan infection [67]; identification of novel allergens [68]; and characterization of the potential allergenicity of transgenic and non-transgenic fish [69]. Proteomics also succeeded in retrieving protein and peptide markers for authentication of aquaculture products, especially fish and crustaceans [70,71,72].

Top-down proteomics arises as a new technology in aquatic sciences. TDP could be rapidly integrated with several research topics in which further information is required of the proteome, namely in the domains of protein polymorphisms and PTMs’ characterization.

Glutathione s-transferases (GSTs) are a family of enzymes that participate in phase II of xenobiotic metabolism, catalyzing the covalent binding of the tripeptide glutathione to xenobiotic compounds, making these compounds less reactive with cellular metabolism and thereby less toxic. These enzymes have been related to functional aspects of the defense response, and adaptation to various water pollutants, in fish and marine and freshwater bivalves. For this reason, GSTs are of particular interest in aquatic sciences and environmental surveillance by functioning as molecular markers of the physiological condition of these organisms, and may indicate the presence of contaminants in the aquatic environment. Proteomic profiling of GSTs, revealed by 2DE and MALDI-TOF/TOF MS, showed several isoforms differing in abundance, molecular mass, and isoelectric points [73]. Proteomic profile of GSTs is different among freshwater and marine bivalves, and also among natural populations separated geographically [74]. Moreover, GST expression is dynamic and living ambient conditions have been shown to determine the number and abundance of expressed isoforms [74,75]. In this context, the elucidation of the structural differences in GST isoforms could help to clarify the functional specificities of this family of enzymes in relation with its substrates and contaminants in the water. The question remains whether all reported isoforms have cellular functions, and how many genes codify the expressed isoforms. In this sense, TDP technology could have a primary role on GST research in aquatic sciences by providing increased protein fragmentation and sequence coverage capacity, thus enabling the identification of the possible differences in amino acid sequences between isoforms or PTMs that could account for the diversity of GSTs in aquatic invertebrates. It is hoped that this elucidation of the proteomic profile of GSTs, leads to a clarification of the molecular functions of the enzyme in relation with the aquatic environment and the multitude of pollutants. Alongside with GSTs there are other protein families which play a relevant function in animal adaptation to the external environment and thus are important molecular markers in aquatic sentinel species. Metallothioneins (MTs) are among this important group of protein markers. Functionally, MTs chelate metals through the thiol group of its cysteine residues and contribute to reducing the toxicity of different types of metals such as cadmium, mercury, silver, and arsenic. Although MTs are codified by different genes, the proteomic profile of this family of proteins seems to be more complex than the number of genes would suggest. Hence, besides amino acid sequence differences, there are possibly conformational modifications and PTMs contributing to the expression of MT isoforms. Leung et al. (2014) [76] verified that MTs’ response in the sentinel green-lipped mussel *Perna viridis* to cadmium (Cd) and hydrogen peroxide (H_2_O_2_) challenges is isoform-specific, which suggests that there are important biochemical and physiological specificities at the isoform level of MTs. Again, TDP can play a role in the characterization of each MT isoform and further reveal the structural and biochemical specificities of the different MT isoforms in relation with metal-binding in environmentally exposed bivalves.

Also in reference to the group of bivalve mollusks is the unique functional interpretation allowed with TDP to the specific gene expansion and molecular diversity that was recently put in evidence by whole-genome sequence analysis. Gene expansion constitutes a unique evolutionary adaptation of bivalves to a sessile lifestyle in the intertidal zone [77,78]. Among the genes present in bivalves with high sequence and structural polymorphisms are the immune and stress response proteins, such as C1q domain-containing proteins and heat shock protein 70 kDa [77,79]. TDP could be employed for a proteomic view and a functional understanding of the encountered genetic polymorphism.

Allergenicity in fish food has been increasingly investigated in the field of aquatic sciences and aquaculture, owing to the food and health safety concerns raised among consumers of fish food, alongside the interest to attract fish-food consumption among the human population. Allergic reactions are attributed mainly to two families of proteins, the fish parvalbumins (PRVBs) [80]—which are calcium-binding protein albumins with a low molecular weight (typically 9–11 kDa) and localized in fast-contracting muscles, brain, and some endocrine tissues—as well as TPMs in crustaceans and mollusks [81], which are functionally integral components of actin filaments. Due to their polymorphic characteristics, PRVBs have been analyzed as molecular markers in the identification of edible species and authentication of fish-food products [82]. It has been found, however, that fish-growth conditions, farming system, and fish diet affects the polymorphism of PRVBs [83] and affects the allergenic potential of the species and the fish-meat products. Although relatively thermostable, the protein can undergo conformational changes at high-temperature treatment, which reduces the protein reactivity to human IgEs [84]. With regard to allergenic TPMs, the investigation showed that heating of shellfish meat enhanced recognition of multiple protein variants in the analyzed shellfish species, pointing to a possible alteration of the protein allergenic potential with this method of fish-meat treatment [81]. In contrast, evidence from protein profiling indicated that high-pressure steaming leads to protein degradation and reduces the levels of TPM in shrimp (*Penaeus monodon*), and consequently reduces the allergenicity in shrimp meat [85]. Not much is actually known with regard to the structure of the reported PRVB isoforms or TPM variants in fish and crustaceans or the structural/conformational differences that mediate the reactivity to IgEs. TDP can have a unique role in this area of research, first aiding the identification of the PRVB isoforms and TPM variants, and then revealing the most relevant epitopes of these proteins that are recognized by human IgEs and trigger allergenic reactions. With this knowledge in mind, farming systems and fish-meat-processing methods could be specifically designed to modulate the expression of PRVBs and TPMs in commercial species to reduce the allergenic potential of the fish meat and increase consumer acceptance.

Although only a few examples of areas of aquatic science research were presented that could integrate TDP approaches, TDP can be employed in many other research topics that seek more information about the proteome, and specifically the polymorphism of the proteome and post-translational protein regulation.

## 5. Conclusions and Future Perspectives

Over the last two decades, proteomics, as other postgenomic tools (e.g., transcriptomics or metabolomics) has become increasingly more common for use in a broad variety of life science fields. Adopting proteomic principles and techniques for use in animal and aquatic sciences has been slower than in other fields, which are traditionally more dynamic and have more access to research funds. Late adoption by AASP researchers has happened in the past with several versions of the 2DE technology (silver nitrate, colloidal Coomassie, fluorescent dyes, and DIGE) and later with the advent of MS-based approaches, particularly shotgun proteomics. Several factors may have contributed to this late technology adoption. Possibly the most relevant can be linked to a generalized lack of knowledge and perception of the principles or possibilities of proteomics research by animal and aquatic sciences researchers. Another important factor is the lack of access or links to proteomic platforms, as very few animal or aquatic science research institutes and universities could afford to purchase their own MS instruments and hire specialized staff for maintenance and operation. Even if the first two difficulties previously mentioned were overcome, traditionally, AASP researchers have always had some difficulty in accessing the high funds needed for a proteomics component in a research project. These difficulties have, however, been curbed over the last decade, thanks to important networking and dissemination efforts by research consortia such as FA1002–Proteomics in Farm Animals COST action (http://www.cost-faproteomics.org/) or the PRIME-XS initiative (http://www.primexs.eu/). These projects were crucial for the development of proteomics in areas such as animal and aquatic sciences; ultimately, allowing researchers in these two fields to get acquainted and become active users of proteomic techniques with important effects on their scientific outputs benefits both science and society as a whole.

A similar trend seems to be in motion for TDP. A literature search such as the one described above reveals that, today, only a handful of researchers within the AASP community are actively using TDP. However, the results obtained and their significance indicate that TDP will likely become more and more common in AASP research. TDP has numerous advantages, the most important is undoubtedly the appropriateness to the study of intact proteins and PTMs that are in turn crucial to understand a specific biological process of key relevance to animal or aquatic sciences.

Furthermore, the existing publications in these two fields clearly demonstrate the high complementary nature of TDP and how it effectively interacts with more classical proteomics approaches such as 2DE or shotgun proteomics. The take-off of TDP in AASP, therefore, seems to have started recently, and it is likely that in the next decade the approach will become much more common in AASP, either alone or as a complement to other proteomics approaches and to other omics-based studies. Its applicability is thus likely to expand to all areas of animal and aquatic sciences, particularly those where PTMs play a crucial role. Accordingly, processes like the study of sperm maturation, tissue, and embryo development, the development of allergies, or the study of specific cellular metabolism are among the areas that would likely benefit from the inclusion of TDP in a research project. The accumulated experience in the past with other proteomics techniques means that possibly a broad inclusion of TDP in animal and aquatic sciences studies will likely be quicker than in the past. However, and as before, collaborations between animal or aquatic sciences researchers and proteomics and mass spectrometry platforms will be of paramount importance.
